# The experience of women with an eating disorder in the perinatal period: a meta-ethnographic study

**DOI:** 10.1186/s12884-018-1762-9

**Published:** 2018-05-02

**Authors:** Sarah Fogarty, Rakime Elmir, Phillipa Hay, Virginia Schmied

**Affiliations:** 1 School of Medicine, Western Sydney University, Locked Bag 1797, Penrith, NSW 2751 Australia; 2 Affiliate Ingham Institute for Applied Medical Research, Centre for Applied Nursing Research (CANR), Liverpool, NSW 2170 Australia; 3 School of Nursing and Midwifery, Western Sydney University, Locked Bag 1797, Penrith, NSW 2751 Australia; 4 School of Medicine and Centre for Health Research, Western Sydney University, Locked Bag 1797, Penrith, NSW 2751 Australia

**Keywords:** Pregnancy, Eating disorders, Anorexia nervosa, Bulimia nervosa, Perinatal, Midwives, Qualitative research, Meta-synthesis, Women

## Abstract

**Background:**

Pregnancy is a time of enormous body transformation. For those with an eating disorder during pregnancy this time of transformation can be distressing and damaging to both the mother and the child. In this meta-ethnographic study, we aimed to examine the experiences of women with an Eating Disorder in the perinatal period; that is during pregnancy and two years following birth.

**Method:**

A meta-ethnographic framework was used in this review. After a systematic online search of the literature using the keywords such as pregnancy, eating disorders, anorexia, bulimia, binge eating disorder, perinatal, postnatal and post-partum, 11 papers, involving 94 women, were included in the review.

**Results:**

A qualitative synthesis of the papers identified 2 key themes. The key theme that emerged during pregnancy was: navigating a ‘new’ eating disorder. The key that emerged in the perinatal period was return to the ‘old’ eating disorder.

**Conclusion:**

Following a tumultuous pregnancy experience, many described returning to their pre-pregnancy eating behaviors and thoughts. These experiences highlight the emotional difficulty experienced having an eating disorder whilst pregnant but they also point to opportunities for intervention and a continued acceptance of body image changes. More research is needed on the experiences of targeted treatment interventions specific for pregnant and postpartum women with an eating disorder and the effectiveness of putative treatment interventions during this period.

**Electronic supplementary material:**

The online version of this article (10.1186/s12884-018-1762-9) contains supplementary material, which is available to authorized users.

## Background

Eating disorders are associated with the highest mortality and morbidity of any mental illness and it is estimated that around 10% of the general population is affected by eating disorders and related behaviors [1, 2]. The most common and well-recognised eating disorders are anorexia nervosa, bulimia nervosa, and binge eating disorder (BED) [3]. For individuals with anorexia nervosa, the state of self-starvation commonly gives a sense of achievement, self-worth and control [3]. Depression and anxiety disorders are common co-morbidities of anorexia nervosa which can make treatment and recovery more difficult [4–6]. Bulimia nervosa and BED both involve binge eating (uncontrolled episodes of overeating) and in bulimia nervosa these are followed by compensatory behaviours such as purging (self-induced vomiting, diuretics or laxatives) or excessive exercise [3]. Due to the menstrual dysfunction that can occur with eating disorders, it was originally considered rare for pregnancy to occur in this population [7, 8]. A growing body of evidence has contradicted this belief and provided confirmation that not only can pregnancy occur during an eating disorder but that it is more common than previously thought [9, 10].

Pregnancy is a time of enormous body transformation. Body and body weight dissatisfaction can affect pregnant women with or without an eating disorder [11]. Women with an eating disorder or a past history of an eating disorder are more vulnerable to heightened body image concerns and the adjustments of an enlarging abdomen and other physical and hormonal changes over which they have no control [12]. There is no uniform behaviour that occurs when a woman with an eating disorder discovers she is pregnant. For some women, their concern for their unborn child motivates a complete cessation of eating disorder behaviours during pregnancy [13–16], yet for other women, the most they can manage is a reduction of disordered eating behaviours [8, 16, 17] and for some their eating disorder stays the same, gets worse or improves for example, from binge-purging to just binging alone [16, 18]. The importance of improving eating disorder behaviour in pregnancy has been highlighted by the link to eating disorders and poor maternal and neonatal outcomes including miscarriages, significant morbidity and increased mortality, pre-eclampsia, and low birth weight [8, 19, 20]. Eating disorders have also been linked to poor health outcomes for pregnant and postpartum women including depressive symptoms during pregnancy, postnatal depression, and poor infant attachment or maternal bonding [21–25].

Lack of disclosure of an eating disorder by women in pregnancy has been reported [26] potentially increasing adverse health outcomes if women do not receive treatment, or assistance to address the eating behaviour symptoms and/or pathology. The need for health professionals providing antenatal care to be more aware of eating disorders in pregnancy and have the capacity and resources to adequately address and treat pregnant women with an eating disorder was reported in a recent qualitative review [13]. It is important to investigate the experiences of women in order to understand how to assist women to reduce disordered eating behaviours during pregnancy, and improve their access to appropriate community, medical and psychological support during the perinatal period. In this perinatal period, research has found that for some individuals their eating disorder symptoms either worsened or returned to their pre-pregnancy levels [8, 11, 25]. This was commonly motivated by the desire to return to a pre-pregnancy weight and shape [24, 27–29]. Returning to eating disorder behaviours in the postpartum period may result in a shorter duration of breastfeeding and may impact on the interaction the mother has with her baby and her relationship with her partner [5, 25, 30]. A recent review on women’s’ experiences of pregnancy and eating disorders found that women experienced turmoil related to fear and guilt about their changing soma and the sense of self, wanting to be a good mother and concern about how others would perceive their behaviour during their pregnancy, however this review did not explore the period following birth [13]. Thus there is need not only to conceptualize the experiences of pregnancy for those with an eating disorder in the antenatal period, but also during the immediate postpartum period and up to the infant age of two years.

There has been an increase in the number of in-depth qualitative studies of women’s experiences of eating disorders in pregnancy and following birth. This offers a timely opportunity to undertake a synthesis of these studies. This meta-ethnographic study aimed to explore the experience of women with an eating disorder or past history of an eating disorder during the perinatal period.

## Methods

A meta-ethnographic framework outlined by Noblit and Hare was used to synthesise the findings reported in included papers as this approach is “well suited to producing new theories and conceptual models” [31]. Meta-ethnography is an interpretive approach combining findings of qualitative studies. This method provides a more comprehensive analysis of the original research findings and provides new insights to the research while simultaneously preserving the authentic meaning of primary data [32]. In this approach there are seven steps to guide the synthesis, see Table 1 [33]. The authors followed the enhancing of transparency in reporting the synthesis of qualitative research (ENTREQ) in reporting this meta-ethnographic study [34]. An ENTREQ checklist is available in the Supporting Information Additional file 1.

**Table 1:** The 7 steps of the Noblit and Hare meta-ethnographic method [33]

Steps		
Step 1	Getting started	These steps involve defining the search criteria and parameters, conducting the search, including, quality assessment and excluding studies [33].
Step 2	Confirming the initial interest	
Step 3	Reading studies	
Step 4	Extracting date from included studies	This step involves extracting data from the included studies and ‘determining how studies are related’ and identifying common themes, and concepts [33].
Step 5	Translating Studies	This step involves ‘comparing first and/or second order themes against each other [33]
Step 6	Synthesising translations	This step creates new third order themes from first and second order concepts.
Step 7	Expressing the synthesis	This step the authors expressing the synthesis implications in relation to clinical practice, policy, treatment, program development and/or research [33].

A comprehensive, pre-planned search strategy was used to seek all available studies. This meta-ethnographic study considered studies and literature that investigated the experiences of women with an eating disorder or past history of an eating disorder during the perinatal period (the perinatal period is defined as from conception to two years post birth). Eating disorders included Anorexia Nervosa Bulimia Nervosa, BED, PICA, Avoidant/Restrictive Food Intake Disorder or Other Specified or Unspecified Feeding or Eating Disorder (OSFED or UFED) [35]. OSFED/UFED replaces the former Eating Disorder Not Otherwise Specified category) [35].

This meta-ethnographic study did not review publications or literature assessing the side effects or outcome of pregnancy on those with an eating disorder or the outcomes for the children. Literature published after 1980 was considered for inclusion in the study. It was important to the authors that the accounts of women’s’ experiences with pregnancy and eating disorders was inclusive. Purely quantitative studies were excluded, as the purpose of meta-ethnographic studies is to synthesize qualitative research findings. The research on the challenges of having an eating disorder during pregnancy was first reported in the late 1980’s. Research published in the languages English, Portuguese or Spanish were also considered for inclusion, as the authors had access to translational services in Portuguese and Spanish.

The meta-ethnographic study considered qualitative papers and mixed methods studies that reported rich qualitative data. This meta-ethnography also considered case studies reporting the experiences of individual women.

**Fig. 1:**
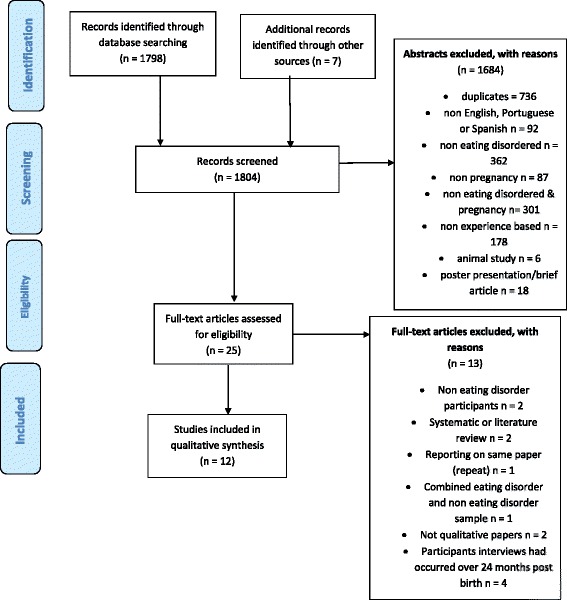
Flow chart of study selection

The first three steps involved in the meta-ethnographic framework outlined by Noblit and Hare [31] involved defining the search criteria and parameters, the search itself, including, quality assessment and excluding studies. Relevant publications were identified using a systematic online search of CINAHL Plus with Full Text (EBSCOhost, EBSCO), Scopus (Pubmed), Dissertation and Theses, Google Scholar and Google up to the date of 14th March 2016. The keywords “**pregnancy**” AND “**eating disorders**”) as well as (“**pregnancy**” AND “**anorexia**”), (“**pregnancy**” AND “**bulimia**”), (“**pregnancy**” AND “**EDNOS**”), (“**pregnancy**” AND “**PICA**”), (“**pregnancy**” AND “**UFED**”), (“**pregnancy**” AND “**OSFED**”), (“**pregnancy**” AND “**binge eating disorder**”), (“**prenatal**” AND “**eating disorders**”), (“**postnatal**” AND “**eating disorders**”), (“**antenatal**” AND “**eating disorders**”), (“**antepartum**” AND “**eating disorders**”), (“**perinatal**” AND “**eating disorders**”), and (“**post-partum**” AND “**eating disorders**”), were used. The Scopus search used ‘All Fields’ and retrieved 1798 results and a further 7 results were retrieved from Dissertation and Theses and a manual search of the reference sections of the identified studies (see Fig. 1). The aim of the search was to obtain sufficient articles to reach ‘conceptual saturation’ from both published and unpublished studies [31]. Author SF undertook identification of the studies. Figure 1 shows the selection of included studies. 1798 publications were initially identified, of which 736 duplicates were removed. Relevant studies were then selected using a two-stage screening procedure.

### First stage screening

The first stage of screening involved eliminating abstracts/publications that did not meet the meta-ethnographic study inclusion criteria based on titles. SF then assessed all literature for possible retrieval of the full text article based on the study inclusion criteria. This first stage of screening generated 25 relevant papers.

### Second stage screening

The 25 articles were read in full by the authors, 12 articles were included and 12 were excluded. There was one instance where two papers used the same data; Burton et al. 2014 [36] and Burton et al. 2015 [20]. In this case, the later of the two papers was used in the review as this represented the most comprehensive dataset. Two papers reported from the same research project; Stringer et al. [37] and Tierney et al. 2011 [8], are treated together within the review. Two articles sampled non-eating disorder participants [38, 39] and one included a combined sample of non-disordered eating participants and participants with disordered eating [40]. Two articles were review articles [13, 41] and two were not qualitative papers [42, 43]. Four papers were excluded as the participants’ interviews had occurred past 24 months following birth [25, 44–46]. All authors were involved in the second stage screening and all authors agreed on the final papers to be included and excluded.

#### Quality appraisal

There is no consensus on which quality assessment tool should be employed for qualitative synthesis and no agreement on how quality assessment tools should be used or applied. There is also a debate about whether quality appraisal should form part of a meta-ethnography at all [32]. The authors used a standardised critical appraisal instrument called Critical Appraisal Skills Programme (CASP) [47] to assess the range of quality of the publications selected for inclusion in the study. The CASP is a qualitative assessment tool that provides an indication of the trustworthiness and relevance of findings (“Critical Appraisal Skills Program (CASP) Qualitative Research Checklist 31.5.13,”). The CASP was completed independently and any disagreements that arose between the reviewers regarding inclusion were resolved through discussion**.** As per the instructions, the CASP was not scored [47]. Similar to Atkins et al., a decision was made that no study that meet the basic criteria for inclusion would be excluded as the CASP provides a description of the range of quality found in papers and major gaps in reporting, if any, not a reproducible guidelines on how to accurately assess good quality studies or when to include or exclude papers.

#### Data extraction

Data extracted included specific details about the participants, study methods, and specific objectives. Step four involved extracting data from the included studies and ‘determining how studies are related’. Authors SF and RE read and re-read the included papers to identify common themes and concepts. The themes reported by authors in each of the included qualitative studies, were extracted as first and second order data. The discussion section of the papers was also read to identify any further key themes or concepts extracted as second order data. The case studies did not present findings in terms of succinct themes. In these cases author SF read the case study results and discussion, and undertook a thematic analysis of the data to include in the review. Authors SF, RE and VS confirmed the first and/or second order data and themes.

#### Data synthesis

**Fig. 2:**
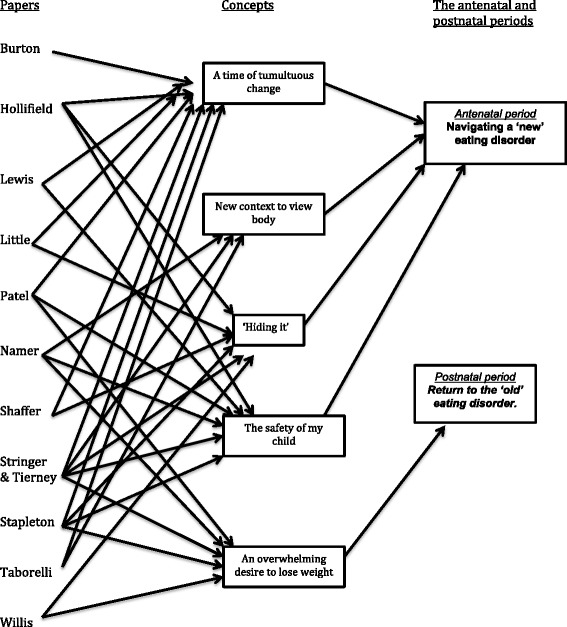
Reciprocal and line of argument concepts

Step five was ‘translating studies’ which compared first and/or second order data against each other. Step six was ‘synthesising translations’. The authors created new third order themes from first and second order concepts [33]. The authors used both reciprocal and refutational translation to note similarities and differences in findings. The majority of the concepts reflected similar findings and themes across the included studies. There were no papers that refuted the findings of another study. This continual process of comparison and development of themes enabled the development of third order themes. The final synthesis included a line of argument that summarized the key findings from the different stages of the antenatal and postnatal periods. See Fig. 2. These findings were presented as a series of themes supported by quotes from the original papers. Again all authors were involved in the step five and six. Step seven was ‘expressing the synthesis’ where the authors reported the implications of the findings of this synthesis for clinical practice, policy, treatment, program development and/or research. The process of synthesis was inductive.

## Results

### Study characteristics

Detailed characteristics such as the populations, study methods, and specific objectives are described in Table 2. The majority of the studies were from the United States (12 studies) and the United Kingdom (9 studies) with the remaining studies being from Australia and South Africa. All studies were published between 1986 and 2015. Sample sizes ranged from 3 to 20 (median 6) across all studies. The total number of women involved across all included studies was 94. The age range for participants in the studies ranged from 21 to 45 years. Twenty-two women were pregnant at the time of the interview and 72 had given birth. The types of eating disorders examined in the study included 22 with Anorexia Nervosa, 17 with Bulimia Nervosa, 10 with Bulimia Nervosa, BED or Eating Disorder Not Otherwise Specified, 34 with an Eating Disorder (type not specified), and 11 with Anorexia Nervosa or Bulimia Nervosa.

**Table 2:** Characteristics of included papers/literature

Author (Year)	Country Setting	Participants N, age, ED features	Study aim/objective	Study design/methodology & transparency	Rigour of analysis and reporting
Journal Articles					
Burton et al. 2015 [20]	Perth, Australia.	20 women, aged between 21 and 40, with an eating disorder diagnosis and who had birthed within the last 12 months. 8 had Anorexia Nervosa(AN), 4 had Bulimia Nervosa (BN), BED or Eating Disorder Not Otherwise Specified (EDNOS). Other 12 not accounted for re eating disorder (ED) diagnosis. All had given birth.	*Aim:* To address a gap in current literature of how pregnant women with eating disorders make meaning of their experience.	*Study design:* Semi-structured in-depth interview. *Methodology:* Used purposive sampling. *Data Collection:* Taped interview, which was then transcribed. *Data analysis:* Colaizzi’s method.	Nothing about those who chose not to take place in the study. Limited data on the critical examination undertaken or the researchers role in the analysis.
Patel et al. 2005 [27]	United Kingdom.	6 mothers, mean age (range 29–42) with either Diagnostic and Statistical Manual of Mental Disease IV (DSM-IV) BN or EDNOS and 9 mothers, mean age 33.6 (range 28–43) at risk for an eating disorder (high concerns about body weight and shape but little behavioural disturbance). Comparison group: 6 mothers, mean age 32.5 (range 28–36), with low body shape and weight concerns. All had given birth.	*Aim:* To examine how three groups of women with different levels of eating disorder psychology, perceived and coped with changes in eating and body shape and weight following pregnancy and the birth of a baby.	*Study design:* In-depth interview. *Methodology:* Used purposive sampling. *Data Collection:* Interview, which was then transcribed. *Data analysis:* Thematic content analysis.	Nothing about those who chose not to take place in the study. Limited data on the critical examination undertaken or the researchers role in the analysis. Nothing in the reporting about receiving ethical approval.
Shaffer et al. 2008 [28]	USA	10 women with a self-reported history of an eating disorder during pregnancy, age range 26–39. 6 were pregnant at time of data collection. 4 had given birth.	*Aim:* To explore the experiences of women who have an eating disorder prior to, or during pregnancy.	*Study design:* In-depth interview. *Methodology:* Used purposive sampling. *Data Collection:* Interview, which was audiotaped then transcribed. *Data analysis:* Manen’s Thematic content analysis.	Nothing about those who chose not to take place in the study.
Stapleton et al. 2008 [29]	United Kingdom.	16 women who self-reported as having an eating disorder, age range 23–44 years. 5 were pregnant at time of data collection. 11 had given birth.	*Aim:* To examine participants’ motivation for, and understandings of, infant-feeding decisions and practices.	*Study design:* In-depth interview. *Methodology:* Used purposive sampling. *Data Collection:* Interview, which was audiotaped then transcribed. *Data analysis:* Feminist and ethnographic approach.	Limited data on the critical examination undertaken or the researchers role in the analysis
Taborelli et al. 2015 [30]	United Kingdom.	12 women with severe eating disorders during pregnancy (8 with Anorexia Nervosa Binge-purge (AN-BP) and 4 with BN), age range 23–39. All had given birth.	*Aim:* To examine in depth the individuals’ experience of transition from pregnancy to motherhood, among women with current eating disorders, focusing on differences between first and subsequent pregnancies.	*Study design:* In-depth interview. *Methodology:* Used purposive sampling. *Data Collection:* Interview, which was audiotaped then transcribed. *Data analysis:* Interpretative phenomenological analysis.	No information on dropouts during the data collection. Limited data on the critical examination undertaken or the researchers role in the analysis
Stringer et al. 2010 [37] and Tierney et al. 2011 [8]	United Kingdom.	8 women with self reported AN or BN, mean age 29.4 years. 3 were pregnant at time of data collection. 5 had given birth.	*Aim:* To examine in depth the experiences of pregnant women with an ED and during the early years of the child’s life.	*Study design:* Semi-structured interview. *Methodology:* Used purposive sampling. *Data Collection:* Interview, which was audiotaped then transcribed. *Data analysis:* Framework analysis.	Limited data on the critical examination undertaken or the researchers role in the analysis
Willis & Rand 1988 [55]	USA.	4 women who meet the criteria (DSM-III) for BN. All women had given birth.	*Aim:* To describe pregnancy outcome in four bulimic women.	*Study design:* Interview. *Methodology:* Used purposive sampling. *Data Collection:* Interview. *Data analysis:* Unclear.	Age of women not reported. Limited information on how data was collected and no information on how the data was analysed.
Case Studies					
Hollifield & Hobdy 1990 [18]	USA.	3 women with DSM diagnosis of BN, aged 23–31, from an eating disorder therapy group who became pregnant. All pregnant at time of interview.	Aim: To present the experiences of three pregnant women with BN whose experience of pregnancy did not match the current literature.	*Study design:* Case studies. *Data Collection:* Semi-structured interview.	No information on the duration or the type of questions asked during the interview.
Lewis & le Grange 1994 [53]	South Africa.	6 mothers aged 27–45 years, with a DSM-IV diagnosis of BN. All had given birth.	*Aim:* To investigate retrospectively i) The emotional experience of pregnancy by women suffering from BN, and ii) Whether pregnancy has an ameliorating, neutral or exacerbating effect on bulimic symptoms during, and/or following, pregnancy.	*Study design:* Case studies. *Data Collection:* Semi-structured interview.	
Little & Lowkes 2000 [52]	USA	3 women with anorexic-bulimic symptoms aged 28–36 years. Two had given birth and one was 9 months pregnant.	*Aim:* Not stated.	*Study design:* Case studies. *Data Collection:* Semi-structured interview.	Not stated what eating disorder the participants were suffering from. The aim of the study not clearly stated.
Namer et al. 1986 [50]	USA	6 women, mean age 28.7 years, with AN. Four were pregnant at time of data collection. Two had given birth.	*Aim:* To learn more about the psychological as well as physiological aspects of pregnancy, birth and the post-partum period in AN.	*Study design:* Case studies. *Data Collection:* Semi-structured interview.	

#### Quality of included studies

The majority of the studies included in the synthesis were of good quality; however, no studies meet all the CASP criteria. Through our critical appraisal we identified several areas where the reporting and methodology was good including study aims, justification of methodology, participant inclusion, data collection, analysis process, and study findings. Areas identified that were not as well reported included a lack of data about those that chose not to take place in the study and limited data on the critical examination undertaken or the role of researchers in the analysis (see Table 2). The case-studies were also generally well reported; however, as expected, there was less description about interview duration, interview questions, sampling method and the role of the researchers in the analysis (17, 46–48). Similar to other authors, the CASP assessment was limited by the papers written report and the word limits of the journals they were published in, which often did not allow for elaboration of the research process [32, 48].

### The experience of pregnancy and the postnatal period for women with an eating disorder

Themes related to pregnancy and the two-year period following birth in the 12 articles are summarized in Tables 3 and 4 respectively. The themes resulting from the reciprocal translation and line of argument across studies are summarized in Table 5. The themes in table three were broken up into time periods: during the pregnancy, during and post pregnancy and the post partum period.

**Table 3:** First and second order themes related to the experience of pregnancy for those with an eating disorder as reported in the primary paper

Publication	Theme 1	Theme 2	Theme 3	Theme 4	Theme 5	Theme 6
Journal Publications						
Burton et al. 2015 [20]	“The battle” between the eating disorder, your body and the baby.	“Going around on the treadmill” – dealing with the eating disorder and the pregnancy and doing the same thing all the time; exhaustion.	“Recreational show ride”- highs and lows and feeling of being out of control.	“Walking the tightrope”- Staying in control and stopping from falling.	“Teetering on the edge”- Feeling like about to fall off into the unknown.	“Uninvited visitor from the past” – Known but unwanted visitor (ED) returning.
Patel et al. 2005 [27]	Loss of pre-pregnancy self - Concerns about pregnancy weight gain - Fear of not losing pregnancy weight	Life transitions - Using weight to control their lives - Physical changes in pregnancy reawakened weight and body concerns	Relationship with family members - Lack of comfort with reliance on help from partners.	Role within wider society - Experiencing the world as hostile and critical in relation to their new maternal selves especially when their was distress about their changed bodies.		
Shaffer et al. 2008 [28]	A constant mental battle to prevent losing control - Struggle to accept weight gain and size - Struggle to accept the pregnancy - Fear of control over their changing bodies.	A distorted body image - Difficulty looking in the mirror - Use of restrictive eating, compulsive exercise, laxative use or binging and purging and controlling weight, a compensation for low self-esteem.	Hiding their experience - Lied about eating behaviours and exercise - Used pregnancy symptoms e.g. nausea to explain/hide behaviours. - Most didn’t tell their physicians or midwives.	Scale-induced trauma at prenatal visits - Routine weigh-ins at prenatal appointments traumatic. - Fear and anxiety regarding weight gain and being weighted.		
Stapleton et al. 2008 [54]	Fighting to control the urge to restrict or binge-purge.	Lack of disclosure of their eating disorder to maternity health professionals				
Taborelli et al. 2015 [49]	Approaching pregnancy: Not expecting to be pregnant	Early pregnancy A difficult transition a) Highly anxiety provoking b) Loss of control over their body Making space for the baby The sacrifice of the eating disorder identity.	Middle to late pregnancy Assuming the pregnancy identity. - a new body to love.			
Stringer et al. 2010 [37] and Tierneyet al. 2011 [8]	Transforming body and eating behaviours - Concern about body shape - First trimester difficult (“seen as fat” rather than pregnant) - Less stress about body shape when obviously pregnant- not “fat” Concern about weight gain during pregnancy.	Uncertainties about child’s shape - Not wanting their children to be obese - Not wanting them to obsess about food	Emotional regulation - Use of SIV in pregnancy to regulate emotion.	Professional awareness - Lack of empathy (from healthcare professionals) - Information deficits -Language used	Type of care - Early Support - High risk surveillance	
Willis & Rand 1988 [55]	Decrease in binge/vomiting behaviours during pregnancy.	SIV occurred during pregnancy.	Mixed reporting of eating disorders to obstetric/maternal health care providers.			
Case Studies						
Hollifield & Hobdy 1990 [18]	Hiding the Eating disorder from obstetricians and other health care personal.	Lied about specific behaviours to their spouse/therapists/family/friends.	Experienced tremendous fears regarding health and wellbeing of their unborn child.	Profound shame and guilt in relation to their inability to refrain from bulimic practices while pregnant.	Rationalisation of behaviours.	
Lewis & le Grange 1994 [53]	Fear of losing control of their eating and weight during pregnancy.	Fear of damaging their unborn child as a result of their unhealthy eating behaviours.	Fear not enough to stop them from engaging in bulimic behavior.	Anxiety about their ability to cope when their baby was born.	Anxiety about the health of their unborn child.	
Little & Lowkes 2000 [52]	Hiding the Eating disorder.	Difficulty giving up the eating disorder behavior.				
Namer et al. 1986 [50]	Positive benefits- a) Being able to fall pregnant made them feel ‘normal’ and ‘better’ b) Pregnancy pleased their husbands Being ‘forced’ to take care of a baby made it easier to take care of themselves	Change in body image- a) Change in focus from stomach to weight and size of thighs as stomach considered to be the baby. Reliance on husband to provide perspective about their changing bodies and weight gain	Change in eating habits- a) Ability to eat 3–4 meals a day and not skip meals Increased variety in foods, eating foods not eaten in years	Effects on marital relationship- a) Husbands pleased with them but arguments about food intake and distress about their insecurity with their appearances.	Mood states and cognitive concerns during pregnancy- a) Concern about baby’s health b) Irritability/Anxiety/Depression especially during first pounds of weight gain c) Fear body wouldn’t return to pre-pregnancy weight d) Later months of pregnancy diminution of anorexic thinking.	Strong desire not to pass on food obsession to their child.

**Table 4:** First and second order themes related to the experience of post-pregnancy for those with an eating disorder as reported in the primary papers

Publication	Theme 1	Theme 2	Theme 3	Theme 4
Journal Publications				
Namer et al. 1986 [50]	All lost pregnancy related weight gain, some weighted less. All concerned and struggling with their weight. Post pregnancy weight loss “allowed” and given permission to lose weight therefore it was hard to control weight loss.	No longer eating 3–4 meals or quantity of food eaten during pregnancy.	All returned to anorexic thinking within several weeks of giving birth.	Anxiety about breastfeeding. Anxiety about not eating enough to breastfeed. No desire to breastfeed as wanted to be able to lose weight.
Patel et al. 2005 [27]	Loss of pre-pregnancy self - Fear of not losing pregnancy weight. - Distress about changes and lack of return to pre-pregnancy weight.	Feeding relationship with infant - Anxiety and discomfort (need to eat to provide milk). - Using breast-feeding to lose weight.	Relationship with family members - Comments about post pregnancy body by partner viewed as derogatory or critical.	
Shaffer et al. 2008 [28]	Post-partum panic and fear - Concern, fear, worry or panic about their bodies post birth	Preconception with body image	Worsening of eating disorder symptoms after birth.	
Stapleton et al. 2008 [54]	Benefits for not breastfeeding - Choice not to breastfeed to be able to engage in eating disorder behavior.	Perceived positives for breastfeeding - Breastfeeding was motivated by the belief it would help with weight loss. - Increase in caloric expenditure from breast feeding allowed consumption of additional food (used as a compensatory mechanism instead of purging) - Feeling that breastfeeding assuages some of the guilt eating disorder women felt about potential damage done to the child because of the mothers eating disorder.	Bottle-feeding - Desire to bottle-feed to ensure child’s nutritional needs are met.	Distress about post pregnancy shape and weight. Resumption of eating disorder behaviours post pregnancy.
Taborelli et al. 2015 [49]	Loss of the pre-pregnancy body identity, loss of pregnant identity. - Distress at post birth body shape Urge to lose weight increases and is compelling post-birth.	Subsequent pregnancies Less dissonance between eating disorders and pregnancy in subsequent pregnancies.		
Tierney et al. 2011 [8]	Fear of failure - Unable to breast feed due to poor mild supply	Emotional regulation - Return to exercise quickly post birth to help regulate negative emotions		
Willis & Rand 1988 [55]	Majority returned to their pregnancy binge/vomiting levels	Extreme distress about post partum body shape prompted relapse.		
Case Studies				
Little & Lowkes 2000 [52]	Increase of eating disorder behaviours postpartum.

**Table 5:** Third order themes: Translation of themes related to pregnancy across the 12 primary studies

Relates to:	Themes	Paper origin
During the pregnancy	A time of tumultuous change 1) ‘I feel grotesque’: an ongoing struggle 2) feelings of loss of control, 3) a tug-of-war.	Burton 2015 Patel 2005 Shaffer 2008 Stapleton 2008 Taborelli 2015 Stringer 2010 and Tierney 2011 Hollifield 1990 Lewis 1994 Little 2000
	New context to view body 1) Experiencing a positive change in eating habits, and 2) a new body to love	Namer 1986 Taborelli 2015 Tierney 2011
	‘Hiding it’	Shaffer et al. 2008 Stapleton 2008 Stringer 2010 Willis 1988 Hollifield 1990 Little 2000
	The safety of my child	Namer 1986 Patel 2005 Stringer 2010 and Tierney 2011 Hollifield 1990 Lewis 1994 Stapleton 2008
Post pregnancy- mother	An overwhelming desire to lose weight	Namer 1986 Patel 2005 Stapleton 2008 Taborelli 2015 Tierney 2011 Willis 1988 Little 2000

### Findings

The findings relate to women’s’ experience of the physical changes to their bodies, their management of an eating disorder during the perinatal period, and concern about the health of their child. The main theme that emerged for women with an eating disorder during pregnancy was navigating a ‘new’ eating disorder. The key theme that emerged for women with an eating disorder post pregnancy was return to the ‘old’ eating disorder.

### Navigating a ‘new’ eating disorder

The experience of having an eating disorder whilst pregnant was not like their pre-pregnancy eating disorder. The eating disorder during pregnancy was ‘new’ and involved times of tumultuous change including feeling grotesque, a struggle over their changing body weight and shape, new feelings of losing control, and a tug-of-war over their desires to both stop and continue their eating disorder behaviours. Some women also experienced their body in a new and positive context experiencing a positive change in body image and eating habits, and discovering a new body to love especially middle to late pregnancy. Women also encountered new reasons for hiding their eating disorder and new challenges around keeping their child safe. Overall these changes express the difficulty that women with an eating disorder can encounter during pregnancy and the depth to which these feelings, struggles and battles consume and construct the pregnancy experiences of women with an eating disorder.

#### ‘Feeling grotesque’: An ongoing struggle

A cornerstone to an eating disorder is body dissatisfaction, drive for thinness and the effect of weight and shape on self-image and self-esteem [3]. Pregnancy is a time of changing body shape and weight and women in these studies grappled with these changes and felt disgust and dissatisfaction about their changing bodies and they were constantly monitoring their weight gain [8, 27, 49, 50].

*“I was weighing myself lots….it was very difficult the first 3 months….when I became pregnant I hated it (long pause); first because I felt fat, and then because my clothes stopped fitting. It just makes you feel worse. I felt just absolutely grotesque”* [49] *p 6*.

For some women, as their body changed, their thighs and their bottom became more of a focus of body dissatisfaction and disgust and they struggled to look at themselves in the mirror with one participant saying *“when I look in the mirror and I see that my butt has gotten bigger, or that I have cellulite for the first time again since I was seventeen and my butt and thighs…those are my bad days”* [28] *p 26.*

Women felt stressed and anxious over their changing body weight and shape during the pregnancy and experienced a struggle with the weight gained. They often perceived this weight change as “getting fat” or: “feeling fat” rather than the outcomes of pregnancy. Women felt this particularly in early pregnancy as they initially gained weight.

*“…my boobs changed first and automatically I thought ‘oh God I’m getting fat’. It was the first three months, you know, when your body starts changing but not enough to look pregnant. So you look like you’re getting fatter rather than being pregnant. So that bit was difficult, emotionally”* [37] *p 8.*

Some of the participants in the study experienced ‘panic’, ‘distress and ‘hatred’ for their bodies [8, 49, 50] and struggled as “they were the heaviest they had ever been” [8] p 1228. This struggle with gaining weight was not the ‘tug-of-war’ oscillation and competition between two strong feelings i.e. managing the eating disorder and the needs of the unborn child, but rather a constant ongoing, daily struggle and a warring down from dealing with the affects of an increasing changing weight and shape.

*“Those ever present annoying voices in my head that were telling me I was fat were a constant battle for me….It was a constant struggle after that to try and have some control over my body and my weight….”.* [20] *p 128.*

#### Feelings of loss of control

Women with an eating disorder during pregnancy experienced a loss of control. A feature of eating disorders is the provision of a sense of control for the sufferer [51]. In the case of pregnancy the capacity to regulate emotions with the usual methods of exercise, food, purging, binging and or restrictions is constrained and if no other method of control is available then a feeling of being out of control can ensue. Women experienced this lack of capacity to regulate emotions as a struggle where they were eventually overwhelmed/worn down by the consistent chatter of their inner eating disorder voices, and their sense of control was swept away.

*“I knew my body would change and I would have to cope with that, but when it began to do so I was not prepared for the struggle that I would go through in having to battle with the day-to-day changes, which I did not like. I got fatter and fatter and I had to battle with the voices telling me that I had lost control and that I would be fat forever”* [20] *p 129.*

*“Control was a big thing because I felt like I could always control my weight and I guess I lost that control in a way because I did gain weight, which was the very opposite of what I wanted to be doing or several years before I got pregnant. It was scary to be out of control of my body and my weight”* [28] *p 25.*

#### Tug of war

Women experienced a ‘tug-of-war’ struggle to manage the needs of the eating disorder and the unborn child, with the two desires often conflicting during the course of the pregnancy. This struggle was experienced as a tug-of-war, with participants being strong at both ends of their convictions. Participants intensely desired both outcomes: their eating disorder behavior and ‘healthy’ eating for a healthy baby. Whilst participants oscillated between opposing desires, their convictions were strong and resolute. The outcome of this struggle appeared to be a fine-tuning of the two competing behaviors and needs; the eating disorder behaviors and the meeting the needs of their unborn child, which often resulted in a change in eating behaviors rather than a total relinquishing their eating disordered behavior [8, 18].

*“…I struggled you know, dealing with the weight gain, kind of between not likening it and being uncomfortable with it but also knowing that it needed to happen to have healthy babies, so it was kind of a constant struggle”* [28] *p25.*

*“I was terribly anorexic for a while in the beginning of the pregnancy. I was so afraid to get fat. I knew I had to, it was an awful conflict”* [52] *p 303.*

The affirmative ‘new’ experiences of their eating disorder while pregnant included experiencing a positive change in body image and eating habits, and a new body to love especially middle to late pregnancy. Some women experienced a time of positive change in their eating disorder behaviors and acceptance, liking and even loving their obviously pregnant bodies and new shape.

#### Experiencing a positive change in eating habits

In contrast to the struggles mentioned in above, pregnancy was also often a time of positive change in eating habits. Women were prompted to make changes to their eating disorder behaviors by being responsible for the well-being of another dependent person. This generally included moderation or a decrease in severity of eating behaviors, and for a small number of women, a complete cessation of eating disorder behaviors. Some women were able to view these changes as positive and necessary, and in a different context to weight gain for medical or eating disorder treatment purposes [8, 49, 52]. It also became apparent that the rigid rules that individuals with an eating disorder set around certain foods, such as fat, diary, carbohydrates for example, could be relaxed during pregnancy.

*“I didn’t want to gain too much weight, so I still restricted but I ate healthier foods like nonfat yoghurt and vegetables”* [52] *p 302.*

*“I was a total freak about my diet and about fat intake and about exercising before I got pregnant, and it continued somewhat during my pregnancy, but again, I had to let myself gain weight”* [28] *p 25.*

#### A new body to love

As the pregnancy progressed some women with an eating disorder were better able to like and even love their bodies as it became clearer to both themselves and others that they were obviously pregnant not ‘fat’ [8]. The influence of body shape and weight on self-evaluation is a feature of eating disorders [35], and thus weight gain and or being ‘fat’ is often viewed as particularly repulsive and displeasing. The context of being pregnant, not ‘fat’, could be experienced positively by liking or even loving their expanding body, in a way that weight gain experienced in their eating disorder did not [8, 27, 49].

*“I didn’t gain any weight for the first five months and then my doctor got really worried so I began to eat and I gained xx pounds in the last three months. I actually loved gaining weight, I really did”* [52] *p 302.*

*“I didn’t mind how I looked when I was pregnant… I was more comfortable because you have a reason, people will see you pregnant and they won’t think ‘oh my god she’s really fat’ they just think she’s just pregnant”* [49] *p 8*

For some women pregnancy provided an opportunity to experience their stomach/abdomen in a positive way such as one participant who *“loved her bump because it was solid and round*” [8] *p 1228* and another participant who felt her “*stomach is beautiful….nice and smooth…..I am the happiest I have ever been with my body”* [8] *p 1228.*

#### ‘Hiding it’

Women were hesitant to disclose their eating behaviors and eating disorder history to others including maternity care providers (midwives, maternity nurses, nurse-midwives and or obstetricians) and or significant others (friends, partner, husband). This practice ranged from actively hiding their eating disorder and eating behaviors to just not sharing their eating disorder history, as they were never asked.

*“With my first no one asked and I didn’t tell. I was ashamed”* [52] *p 305.*

*“I never shared it with my husband. He thought I had completely stopped purging. I pretty much had stopped it, but there were times I would indulge in a craving knowing that I could get away with throwing up by just saying that I was nauseous and it just came up”* [28] *p 26.*

The lack of disclosure of their eating disorder to healthcare providers had the potential to add to their distress especially when being weighed at antenatal visits.

*“It was always a trauma. It’s very hard. I wish you weren’t facing the scale and they didn’t let you see your weight. I was stressed every time I had to go to the doctor and be weighted to see how much I’d gained because I was very, very rigid so that I wouldn’t gain what I thought was too much weight.”* [28] *p 27.*

#### The safety of my child

Research has shown a link to eating disorders and poor maternal and neonatal outcomes including miscarriages, significant morbidity and increased mortality, pre-eclampsia, low birth weight, and poor infant attachment/bonding [8, 19–25]. Women in these studies were very aware of the capacity of their behaviors to influence the health of their unborn child [8, 18, 27, 50, 53, 54] and they were concerned and worried that their eating behaviours may lead to serious damage to their child during the pregnancy [8, 18, 20, 36, 50, 53].

*“The pregnancy was like a rollercoaster ride. It caught my breath at times and made me feel really scared…..Was it going to conclude safely or would my baby be damaged with the fallout from the ride?”* [20] *p 129.*

*“I am not doing it (eating more) for myself, you understand, but have to do it so that the baby will be O.K.”* [50] *p 842.*

Women commonly expressed their general concerns about their child’s health rather than citing a specific outcome. The included studies did not provide enough information to determine if the mothers were aware of specific risks related to eating disorders and maternal health or just generalised risks.

### Return to the ‘old’ eating disorder

The main theme that emerged for women with an eating disorder following the birth was a return to the ‘old’ eating disorder. During this time women experienced an overwhelming desire to lose weight and a pull to return to the old and comfortable eating disorder behaviours. The overwhelming desire to lose weight involved not only how women experienced their weight and shape after giving birth but it also influenced their feeding choices for their newborn. This led to a distressing and precarious time where women with an eating disorder were highly vulnerable to returning to eating disorder behavior and symptomology.

Women with an eating disorder experienced anxiety and distress about their post pregnancy weight and shape and capacity to lose their pregnancy weight [27, 50, 55]. The postpartum body shape and weight were perceived as repulsive and upsetting, and there was accompanying anxiety and distress [28, 29, 49]. There was also a strong desire and need to lose the pregnancy weight and return to their post pregnancy shape and weight [27, 49]. The majority of the women quickly returned to their pre-pregnancy eating disorder behaviors and thoughts. It was apparent that the changes that had occurred during pregnancy, such as the reduction of eating behaviors, liking their body, disappeared once the baby was born and or after breastfeeding stopped.

*I think the harder part is after you’ve had they baby and coming to terms with that [sic] new body, you know, when you still look like you’re pregnant. I probably had more instances of binging and purging right after the baby was born because of the lack of sleep and also the feeling that, well, the baby’s not inside of me anymore, you know, I’m not really hurting the baby if I binge and purge* [28] *p 27.*

*I joined a dieting group for a week… I didn’t lose anything so I left…I weighed myself all the time waiting for a miracle… I have the intention of doing it (taking appetite suppressants) again as soon as I stop breastfeeding. The only way I know how to control this voice inside of me telling me to eat, eat, eat is when I take those pills* [27] *p 354.*

In the postnatal period, women’s decisions around infant feeding were influenced by their overwhelming desire to lose the pregnancy weight and the effect this may have on their weight and shape [27, 50, 52, 54]. Some women chose to breastfeed because they perceived they would lose weight faster such as *“I think it (breastfeeding) was partly about me behaving selfishly. It was knowing that it brought your figure back more quickly so I kept putting off weaning him because I know the weight was still dropping off me”* [54] *p 111.* For others breastfeeding was avoided so that eating disorder behaviors could resume e.g. exercise, restricting, and or purging.

*I couldn’t breastfeed. I just couldn’t. I was desperate to get rid of the weight* [54] *p 110.*

*As soon as I could walk after I had her, I started doing exercises again… I just wanted to get the fat off. I was going to the gym 2 or 3 hours a day in the morning at 3.30 in the morning until 5.30 in the morning or 2 in the morning until 5. Just to get the fat off. I didn’t even attempt to breastfeed; I was so focused on losing the weight* [52] *p 303.*

## Discussion

The present research is to our knowledge the first meta-ethnographic study exploring the perinatal period for women with eating disorders or a history of an eating disorder. The findings from this study relate to women’s’ experience of the physical changes to their bodies, their management of an eating disorder during the perinatal period, and concern about the health of their child. Pregnancy was experienced as a time of tumultuous change with stress and anxiety over body weight and shape changes, ‘a tug-of-war’ battle’ managing the needs of the eating disorder and the unborn child, feelings of loss of control. For some women they experienced a positive change in body image and eating habits, and found a new way to love their body, especially middle to late pregnancy. The main theme that emerged for women with an eating disorder during pregnancy was navigating a ‘new’ eating disorder. The key theme that emerged for women with an eating disorder post pregnancy was return to the ‘old’ eating disorder.

While Tierney et al. reported similar findings of an internal turmoil [13] they did not report on the potential for positive experiences. In this meta-ethnographic study we found some women experienced a new context to view their body and talked of a new body to love once they were more obviously pregnant and a motivation to alter eating habits [13]. Tierney and colleagues mention: “pregnancy is an optimum moment for women to consider their behaviours and make lasting changes to eating and weight control practices” [13] p. 547, they do not provide evidence of where these opportunities to intervene may occur.

Our ethnographic study findings indicate a number of possible opportune times for intervention. These include during the stage where women experience more acceptance of their body, when their desire is strong not to harm the health of their child, and during the tug-of-war, where the desire to meet the needs of their unborn child is greater than the desire of the eating disorder. In addition to using these opportunities to intervene, there is also the opportunity to work with women to promote a continuation of greater body acceptance and change post pregnancy. While rare in our sample of women, other studies have shown that some women, due to their concern for their unborn child, experience a complete cessation of eating disorder behaviours during pregnancy [14–16]. Taking advantage of the desire of women with an eating disorder to care for their unborn child could be explored through the development of a program that aims to support women to understand the specific risks eating disorder pose to their child and to highlight the positive effects in terms of child development and a stronger sense of connectedness with their mother that the baby might experience from these changes. In addition, it is hypothesized that treatment aimed at capitalizing on this opportunity to intervene and influence post pregnancy behaviors may need to be more intensive than pre-pregnancy treatment given the changing nature of the body during pregnancy and treatments may need to focus more on interventions that provide resources for self-nurturing, reducing anxiety and coping with stress, including a focus on the mother-infant relationship [56].

The time after pregnancy is experienced as a defining moment, it is during this time that women can return or rebound to pre-pregnancy eating disorder levels or more dangerously, experience greater eating disorder behaviors. The changes experienced during pregnancy, which are commonly motivated by the needs of their unborn child [29, 49, 50, 52], are replaced by distress and disgust with their new shape and weight, and the opportunity to return to their pre-pregnancy eating behaviors without the fear of directly effecting their child [27–29, 49]. Whilst positive changes in eating disorder have been experienced during pregnancy [49, 52, 57] these changes are generally not sustained post pregnancy [27–29, 49, 50]. Thus it is imperative to ensure that any mental health intervention/treatment for pregnant women with an eating disorder continues into the postpartum period and the possible ‘red flag times’ within these two periods to better understand how to enhance support and treatment options for pregnant and postpartum women with an eating disorder. Ideally, maternity care providers would also provide continuity of care for women with an eating disorder in the perinatal period, supporting the mother in her transition to motherhood and linking her with services and referral pathways to meet her complex health care needs. Further research is needed to determine the best practice for ensuring continuity of care among women with eating disorders.

Eating disorders are often a secretive disease [58] and this seems to extend into pregnancy with a finding of a lack of disclosure or hiding of eating behaviors to maternity care providers and or significant others. This behavior of hiding the eating disorder, whilst having common features with non-pregnant eating disorder individuals, seems to be distinguished in pregnancy by the motivation of discomfort from receiving inappropriate care and also the shame of having an eating disorder while pregnant although more qualitative research is needed to determine the exact differences. The findings of this meta-ethnographic study revealed the reasons for the lack of disclosure included not being asked by maternity care providers, discomfort disclosing their eating disorder, and a lack of appropriate care, specific for their eating disorder, when they did disclose their eating disorder [18, 28, 29, 55]. The reason for lack of disclosure and barriers to accessing health care treatment for eating disorders is similar among pregnancy and non-pregnant women. These include the severity of the health threat, the perception of the individuals’ eating disorder health, feelings of shame, stigma and the clinicians understanding and capacity to recognize an eating disorder [59]. While women with an eating disorder who are not pregnant may choose not to disclose and seek any medical treatment, pregnant women with an eating disorder are often already seeking care through a maternity care provider and may have frequent visits with a maternity care provider thus providing multiple potential opportunities for disclosure. As such, maternity care providers are in a unique position to provide an environment where pregnant individuals would feel comfortable disclosing their eating disorder. Maternity care providers could also help identify potential eating disorder behaviors, explain how serious eating disorders are especially during pregnancy and provide understanding and support [59]. Improving the eating disorder health literacy of maternal health care providers to be able to provide these services is an integral part of improving disclosure of an eating disorder during pregnancy and utilizing this opportunity to intervene [60].

The lack of disclosure or hiding of an eating disorder during pregnancy also raises a number of implications for antenatal care including screening and screening tools, assessment and support during the antenatal period. There has been a growing emphasis to identify women at risk for emotional and mental health conditions in the perinatal period [61]. In many States in Australia and overseas, the knowledge of the impact of previous or current social and or mental health problems has led to the implementation of psychosocial assessment and depression screening as part of routine clinical practice of midwives and child and family health nurses [62]. However, this study identifies a need to better prepare and train health professionals, such as midwives, child and family health nurses and doctors, for this work and thus support them to be able to offer empathetic and sensitive care to women who are disclosing personal information [62]. The aim of identifying women with risk factors for poor perinatal health including those with an eating disorder is to be able to offer early intervention, support services and appropriate individualized care [13, 61]. The ongoing care by maternity care providers of a woman who has been identified with an eating disorder requires additional skills to screening and assessing. It has been identified that maternity care providers may not have the skills, knowledge, or work resources to manage pregnant women with an eating disorder [13] and their specific needs such as sensitivity when being weighed and managing individuals fears about their changing shape. There is a need, despite the challenges of time and other work demands, to provide the resources for training and eating disorder awareness to maternity care providers.

There is substantial research on the effects of eating disorders on the health of the mother and the unborn baby including miscarriages, significant morbidity and increased mortality, pre-eclampsia, and low birth weight [8, 19, 20] and a growing body of research investigating the experiences of those with an eating disorder during pregnancy [8, 20, 28, 29, 49] however there is one notable aspect of research that is missing and that is research on the efficacy of treatment interventions specifically during the pregnancy period. A brief search of research databases by the authors did not find any studies that address current best-practice interventions for eating disorder treatment or any other adjunct therapies assessing treatment efficacy for those with an eating disorder during pregnancy. While there may be some published research studies available, more research is needed on the effectiveness of a specific perinatal treatment for those with an eating disorder. The publication of well-presented casa studies and case-series that highlight treatment approaches that may be beneficial is required and these studies may provide the foundations for much needed larger efficacy/effectiveness research studies. In addition there was no qualitative research on how treatment was experienced during pregnancy and the aspects of treatment that were helpful and those that were not beneficial. Specific pregnancy related treatment is needed during the perinatal to maximize the opportunity to intervene and make lasting changes, and treatment must address both the turmoil experienced and new and effective ways to manage stress and feeling out of control. Future research into psychological approaches that focus on reinforcing the positive changes during pregnancy, and strategies addressing how these might be carried over once the baby is born is needed. In addition, given the distressing and precarious time that is experienced post pregnancy, qualitative research into a treatment or intervention that specifically aimed to provide support, education and treatment during the post-partum period is warranted.

### Strengths and weaknesses

The meta-ethnographic study undertaken did not lead to a loss of contextualised data about the lived experience of women with an eating disorder during the perinatal period as the context and integrity of the original data was the backbone to the themes. The process of undertaking the meta-ethnographic study highlighted important areas for future research. Understanding more deeply about other factors that may influence the experiences of perinatal women with an eating disorder such as their relationship with the baby’s father, if the pregnancy was planned (or unplanned), if they were first-time mothers, social support received, life stressors, and or if they felt unwell during their pregnancy. The current body of qualitative literature on perinatal women with an eating disorder has limited reporting on if the pregnancies were planned (or unplanned) and they do not generally report on other possible contributing factors. Six papers reported if the pregnancy was planned [8, 18, 49, 50, 53, 55] with 19 of 39 participants having planned their pregnancies. These papers did not report on the impact a planned or unplanned pregnancy had on the perspective or experiences of having an eating disorder during the perinatal period.

Limitations of this study were the inclusion of papers that specifically investigated women with only Anorexia Nervosa or Bulimia Nervosa or papers that investigated eating disorders in general. There were no studies that specifically investigated the experiences of those with BED thus the review findings may not be applicable for those with BED. The meta-ethnographic study limited participation to those interviewed within 24 months of giving birth and papers in English, Spanish and Portuguese, which meant that some papers and themes might have been not identified. The authors acknowledge that the number of studies included (including only 6 studies on the post-natal period) is not large however the role of meta-ethnography is not to summaries the entire body of knowledge but to focus on conceptual insight and “including too many studies might make conceptual analysis ‘unwieldy’ or make it difficult to maintain insight” [63]. The authors feel that the number of papers included were sufficient to produce conceptual insight.

#### Clinical implications

An avenue for addressing maternal care for those with an eating disorder is to target maternity care providers at hospitals with an interest in eating disorders and to provide training, support and a pathway of care specifically to these individuals. Eating disorder treatment providers could then refer pregnant eating disorder patients to these trained maternity care providers (and visa versa) and they could be included in part of the eating disorder multi-disciplinary team, all of whom are working with the same aims and goals for the pregnant woman (59). Pregnant women may see multiple health providers during their pregnancy, such as midwives and doctors, meaning the individual might experience a lack on continuity of antenatal eating disorder care. The importance of the appropriate practitioner in the management and recovery from an eating disorder has been well highlighted (60, 61) and the above mentioned treatment option of specific eating disorder pregnancy related treatment would also provide continuity of care for the pregnant woman with an eating disorder.

There is a need for more training for health professionals such as midwives, child and family health nurses and doctors to screen, and then provide appropriate ongoing support and care, for pregnant women with an eating disorder.

## Conclusion

This meta-ethnographic review provides insight into the experience of having an eating disorder during the perinatal period and highlighted the ‘new’ and tumultuous nature of this. It also drew attention to the difficulties experienced in the post-partum period with a drive for weight loss and a return to pre-pregnancy eating behaviors and thoughts. These experiences highlight the turmoil and crisis of having an eating disorder whilst pregnant but they also highlight the opportunities for intervention and a continued acceptance of body image changes. There are several clinical implications regarding the care of pregnant and postpartum women particularly around opportune times to intervene include during the stage where women experience more acceptance of their body, when their desire is strong not to harm the health of their child, and during the “tug-of-war”, where the desire to meet the needs of their unborn child is greater than the desire of the eating disorder. Further research is needed on effective treatment and management interventions during the perinatal period and the need to educate health care providers who are able to provide care for women with an eating disorder in the perinatal period.

## Additional file

Additional file 1: ENTREQ Checlist. ENTREQ Checklist with page location of each item. (DOCX 102 kb)

## Abbreviations

AN: Anorexia nervosa
AN-BP: Anorexia nervosa binge-purge
BED: Binge eating disorder
BN: Bulimia nervosa
CASP: Critical appraisal skills program
DSM: Diagnostic and statistical manual of mental disease
ED: Eating disorder
EDNOS: Eating disorder not otherwise specified
OSFED: Other specified
UFED: Unspecified feeding or eating disorder
