# Supplementary material for: The experience of women with an eating disorder in the perinatal period: a meta-ethnographic study
Source: BMC Pregnancy Childbirth. 2018 May 2;18:121. doi: 10.1186/s12884-018-1762-9 (PMC5932857; doi:10.1186/s12884-018-1762-9)
Supplement: Supplementary file 1 — ENTREQ Checlist. ENTREQ Checklist with page location of each item. (DOCX 102 kb) [file 12884_2018_1762_MOESM1_ESM.docx]

**Additional File 1: ENTREQ checklist**

| **Number** | **Item** | **Guide and Description** | **In text page number** |
| --- | --- | --- | --- |
| 1 | Aim | State the research question the synthesis addresses. | p. 3 |
| 2 | Synthesis methodology | Identify the synthesis methodology or theoretical framework, which underpins the synthesis, and describe the rationale for choice of methodology. | p. 3 |
| 3 | Approach to searching | Indicate whether the search was pre-planned or iterative. | p. 3 |
| 4 | Inclusion criteria | Specify the inclusion/exclusion criteria. | p. 4 |
| 5 | Data sources | Describe the information sources used and when the searches were conducted; provide rationale for using the data sources. | p. 4 |
| 6 | Electronic Search strategy | Describe the literature search. | p. 4 |
| 7 | Study screening methods | Describe the process of study screening and sifting. | p. 5 |
| 8 | Study characteristics | Present the characteristics of the included studies. | Table 1 &  p. 7 |
| 9 | Study selection results | Identify the number of studies screened and provide reasons for study exclusion. | Figure 1 &  p. 5 |
| 10 | Rationale for appraisal | Describe the rationale and approach used to appraise the studies or selected findings (e.g. assessment of conduct: validity and robustness). | p. 6 |
| 11 | Appraisal items | Sate the tools, frameworks, and criteria used to appraise the studies or selected findings (e.g. CASP, QARI). | p. 6 |
| 12 | Appraisal process | Indicate whether the appraisal was conducted independently, by more than one reviewer and if consensus was required. | p. 6 |
| 13 | Appraisal results | Present results of the quality assessment and indicate which articles, if an, were weighted/excluded based on the assessment and give the rationale. | Table 1 &  p. 14 |
| 14 | Data extraction | Indicate which sections of the primary studies were analysed and how were the data extracted from the primary studies. | p. 6-7 |
| 15 | Software | State any computer software used, if any. | N/A |
| 16 | Number of reviewers | Identify who was involved in coding an analysis. | p. 6 |
| 17 | Coding | Describe the process for coding of data. | p. 6 |
| 18 | Study comparison | Describe how were comparisons made within and across studies. | Table 2a, 2b & p. 5-6 |
| 19 | Derivation of themes | Explain whether the process of deriving the themes or constructs was inductive or deductive. | p. 6 |
| 20 | Quotations | Provide quotations from the primary studies to illustrate themes/constructs, and identify whether the quotations were participant quotations or the authors interpretation. | p. 8-14 |
| 21 | Synthesis output | Present rich, compelling and useful results that go beyond a summary of the primary studies. | Table 3 &  p. 7-14 |
